# Fluorescent labelling of membrane fatty acid transporter CD36 (SR-B2) in the extracellular loop

**DOI:** 10.1371/journal.pone.0210704

**Published:** 2019-01-23

**Authors:** Yilin Liu, Ricardo Rodriguez-Calvo, Shujin Wang, Xiaoqing Zhu, Jos L. V. Broers, Jan F. C. Glatz, Joost J. F. P. Luiken, Dietbert Neumann

**Affiliations:** 1 Department of Molecular Genetics, CARIM School for Cardiovascular Diseases, Maastricht University, Maastricht, the Netherlands; 2 Department of Molecular Cell Biology, CARIM School for Cardiovascular Diseases, Maastricht University, Maastricht, the Netherlands; Michigan State University, UNITED STATES

## Abstract

**Context:**

Upon palmitate oversupply, membrane fatty acid-transporter CD36 (SR-B2) permanently translocates from endosomal storage to the sarcolemma, inducing lipotoxicity. CD36 translocation results from endosomal alkalinisation elicited by palmitate-induced disattachment of the cytoplasmic V_1_-subcomplex from the membrane-integrated V_0_-subcomplex of vacuolar-type H^+^-ATPase.

**Objective:**

Develop a CD36 fluorescent labeling technique as initial step towards live cell imaging.

**Methods:**

Three human CD36 (hCD36) mutants were constructed via insertion of a tetracysteine motif at different positions within the extracellular domain. Constructs were lentivirally transduced for subsequent CD36 labeling with fluorescein-arsenical hairpin-binder (FlAsH). Cell imaging was combined with V_0_/V_1_ immunostaining and Western blotting.

**Results:**

Transduction of hCD36-wildtype and mutants yielded corresponding proteins in HL-1 cardiomyocytes. Tetracysteine mutant-2 (hCD36-TC2) showed similar fatty acid uptake to wildtype. FlAsH staining revealed a speckled pattern reminiscent of endosomes. We found decreased V_1_ co-localization with CD36 upon high-palmitate culturing. Conversely, V_0_ consistently co-localized with CD36.

**Conclusion:**

hCD36-TC2 is a possible candidate for application of biarsenical dyes in live imaging studies pending further investigation. Our data is compatible with V_0_/V_1_ disassembly in high-palmitate-treated cells.

## Introduction

Accumulation of fatty acids and their metabolites in cardiomyocytes is a cause for cardiac insulin resistance, and ultimately leads to cardiac dysfunction, commonly referred to as diabetic cardiomyopathy [1, 2]. CD36, also known as fatty acid translocase or scavenger receptor (SR-B2), is the predominant fatty acid transporter in the heart, mediating an estimated 70% of the cardiac fatty acid flux [2, 3]. Under normal conditions, CD36 cycles between intracellular storage compartments and the sarcolemma. Hormonal or mechanical stimuli induce translocation of CD36 to the sarcolemma to enhance fatty acid uptake and its subsequent metabolism. Thus, CD36 translocation forms the basis of dynamic adaptation of the fatty acid transport rate into cardiomyocytes in response to physiological stimuli [4, 5]. Studies in insulin-resistant rodent models have shown increased abundance of CD36 at the cell surface due to a sustained translocation from endosomes in the absence of changes in CD36 total expression, indicating that fatty acid oversupply induces alterations in subcellular CD36 cycling [6, 7]. Aberrantly high sarcolemmal CD36 presence is seen in association with high extracellular concentrations of long-chain fatty acids such as palmitate. Palmitate causes a chronically elevated influx of fatty acids into cardiomyocytes, which is directly coupled to lipid accumulation, insulin resistance and contractile dysfunction. Thus, permanent CD36 relocation plays a vital role in the development of diabetic cardiomyopathy [6].

Previous studies have identified vacuolar-type H^+^-ATPase (v-ATPase) involvement in the CD36 translocation process [8]. As a multi-unit proton pump occurring in acidic organelles, v-ATPase is responsible for endosomal acidification. V-ATPase consist of two sub-complexes, i.e., the integral membrane sub-complex V_0_ and the cytoplasmic sub-complex V_1_ [9]. Biochemical assays have demonstrated that pharmacologically induced v-ATPase inhibition (using the specific inhibitor Bafilomycin) leads to expulsion of CD36 from the endosomes to the sarcolemma [8]. Biochemical data further suggested that CD36 translocation during lipid overload to the sarcolemma is due to the disassembly of the V_1_ sub-complex into the cytoplasm, resulting in a loss of v-ATPase proton pumping activity and hence a decreased acidification of the endosomes [10].

Live cellular imaging would be an effective approach to visualize the CD36 translocation process. Green fluorescent proteins (GFP) have been widely used for biological imaging to investigate protein localization, intracellular trafficking, and protein–protein interactions [11]. Previous studies have shown the functional discrepancy of N- and C-terminally GFP-tagged hCD36 trafficking in Chinese hamster ovary (CHO) cells [12]. Notably, the N- and C-termini of CD36 are intracellular [2]. As an alternative method, immune labelling offers the potential to capture CD36 in live cells provided the antibodies recognise the extracellular domain. However, such antibodies can also block CD36 mediated lipid uptake [13] and due to their bulky nature can be expected to interfere with other known functions of CD36.

The genetically encoded tetracysteine tag (with amino acid sequence Cys-Cys-Pro-Gly-Cys-Cys) offers the advantage of a small size protein label [14, 15]. As a result, potentially the tetracysteine motif can be introduced within a functional domain of a protein without disrupting its biological function. The tetracysteine tag binds with high-affinity to biarsenical dyes, such as fluorescein arsenical hairpin binder (FlAsH) and resorufin arsenical hairpin binder (ReAsH) [16, 17]. FlAsH or ReAsH are prepared as ethane dithiol (EDT) nonfluorescent complexes. Upon binding of either FlAsH-EDT_2_ or ReAsH-EDT_2_ to the tetracysteine sequence, the displacement of EDT causes FlAsH or ReAsH to become highly fluorescent green or red dyes, respectively.

In the present study, we selected three different positions in the extracellular loop of human CD36 to incorporate the tetracysteine motif as potential FlAsH labelling site. Upon lentiviral transduction we used Western blotting, functional assays and fluorescent microscopy analyses to identify a tetracysteine construct that would not interfere with CD36 functioning. We further investigated the co-localization of v-ATPase V_0_/V_1_ subunits with tetracysteine-tagged CD36, which is expected to be dynamically changing depending on stimuli based on our previous studies.

## Materials and methods

### Plasmid constructs

**Fig 1:**
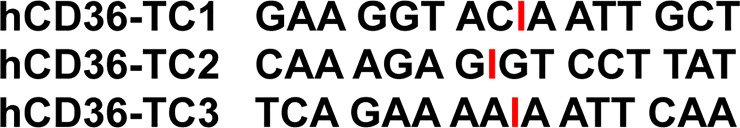
CD36 target sites for insertion of the tetracysteine motif. Part of CD36 coding sequence showing the exact positions where the tetracysteine tag was introduced for each of the mutant.

The tetracysteine motif Cys-Cys-Pro-Gly-Cys-Cys was engineered into the extracellular loop of CD36 by two-step PCR. Three different sites were selected for this insertion, encoded at the following amino acid (aa) positions: hCD36-TC1: aa48; hCD36-TC2: aa89; hCD36-TC3: aa398 (**Fig 1**). To genetically encode the tetracysteine motif at the desired position of myc-DDK-tagged hCD36 cDNA, we designed a set of primers containing the sequence 5’-XXXXXTGCTGTCCAGGATGCTGCXXXXX-3’ and general primers for the sequence of hCD36 **(Table 1)**.

**Table 1:** Primer sequences used for two-step PCR.

	Sense	Antisense
**General primer**	5’—AGGAGATCTGCCGCCGCGATCGCC—3’ **(*Bgl*II)**	5’—CCACCCGGGATCTGTTCAGGAAACAGCTATG—3’ (SmaI)
**hCD36-TC1**	5’—CGAAGAAGGTACTGCTGTCCAGGATGCTGCAATTGCTTTTAA—3’	5’—TTAAAAGCAATTGCAGCATCCTGGACAGCAGTACCTTCTTCG—3’
**hCD36-TC2**	5’—TTAAGCAAAGAGTGCTGTCCAGGATGCTGCGTCCTTATACGT—3’	5’—ACGTATAAGGACGCAGCATCCTGGACAGCACTCTTTGCTTAA—3’
**hCD36-TC3**	5’—GCCATCAGAAAATGCTGTCCAGGATGCTGCAATTCAAGTATT—3’	5’—AATACTTGAATTGCAGCATCCTGGACAGCATTTTCTGATGGC—3’

**Fig 2:**
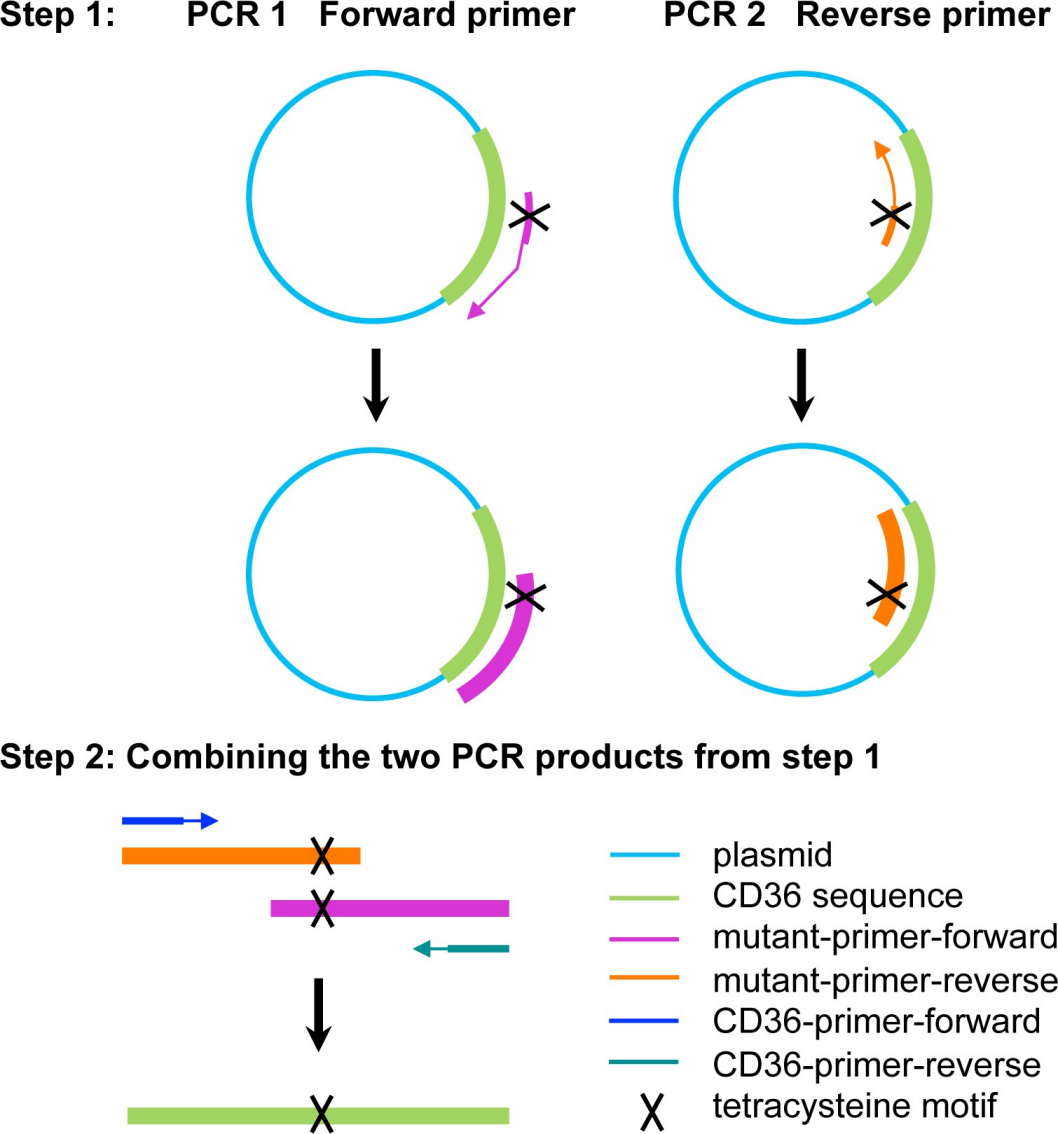
Two-step PCR applied for generation of CD36 tetracysteine tag mutants. For each mutant in step 1 two PCR reactions were performed in parallel either using forward or reverse mutant primers carrying the tetracysteine motif. The products from the first step were applied as templates for the second step. General primers for amplification of the full coding sequence of CD36 generated the entire coding sequence including the desired tetracysteine tag sequence.

The intended mutations were incorporated into cDNA of myc-DDK-tagged hCD36 by two-step PCR (**Fig 2**). Mutants were confirmed by sequencing (Eurogentec, Maastricht, The Netherlands). The three mutant sequences of CD36 were cut from pCMV6 construct (Origene, Rockville, United States) using the restriction enzymes, *Bgl*II/*Sma*I, and subcloned to the lentiviral vector pLVX by ligation to the same restriction sites.

### Virus production and infections

HEK293T cells were plated at 40% confluency in 10 cm dishes. After 24 h, lentiviral vector pLVX-Puro (Clontech, No. 632164), together with the two packaging plasmids, psPAX2 and pMD2.G (Envelope and GagPol), were transfected into 293T cells by using P-PEI. DMEM medium supplemented with 10% fetal calf serum (FCS) and 1% 100 U/ml penicillin-streptomycin was refreshed, 24 h after transfection. The medium was harvested after both 48 h and 72 h, filtered through 0.45 μm filters and used for infection. Mouse HL-1 cardiomyocytes were cultured in Claycomb medium at 60% confluency in 6-well plate as previously described [18]. 3 Hours after seeding, cell culture medium was aspirated, and the cells were infected with viral supernatant in the presence of polybrene (8 μg/ml) overnight. After recovery for 48 h with fresh medium, infected cells were selected with puromycin (4 μg/ml) for 48 h. Thereafter cells were used for experiments as described.

### Western blotting

The lentivirally infected cell lysates were prepared and analysed by Western blotting as previously described [19]. The following primary antibodies were used: anti-hCD36 (1: 20000 in TBST; kindly provided by Dr. Tandon, Bethesda, USA) and anti-DDK (1: 2000 in 5% BSA in TBST; Origene). Primary antibody binding was detected by anti-mouse secondary antibody (1:20000 in TBST; Dako).

### [^14^C] Palmitate uptake assays

The infected different HL-1 cardiomyocytes were cultured on gelatin/fibronectin-coated coverslips in 12-well plates. Prior to the uptake assay, cells were incubated in serum-free depletion medium for 16 h. Then cells were treated either with or without 200 nM insulin for 30 min. Subsequently, [1-^14^C] palmitate (coupled to BSA in a palmitate/BSA ratio of 1:3) was added to a final concentration of 20 μM. After 10 min, uptake was terminated by Stop medium (Serum-free depletion medium, 0.2 mM phloretin). After transfer of the coverslips into new cell culture wells with stop medium, cells were lysed by adding 1 M NaOH and then cell-associated palmitate was measured by scintillation counting of ^14^C.

### CD36 labeling with FlAsH-EDT_2_ and immunofluorescence

HL-1 cardiomyocytes infected with the various CD36 constructs were cultured on gelatin/fibronectin-coated coverslips in 24-well plate. Prior to labelling, cells were incubated in serum-free depletion medium for 16 h. Next day, medium was removed and cells were washed twice with HBSS with 5.6 mM glucose. Cells were incubated with 500 nM FlAsH-EDT_2_ (Thermofisher) in HBSS/glucose medium for 1 h at 37°C. After the aspiration of FlAsH-EDT_2_, non-specifically bound dye was removed by incubation with 250 μM EDT for 10 min at 37°C. Cells were washed seven times with HBSS/glucose and fixed with 4% formaldehyde afterwards for 10 min at room temperature. Subsequently, cells were rinsed twice in PBS and permeabilised by 0.2% Triton X-100 in PBS for 15 min at room temperature, followed by 90 min of incubation with anti-Myc antibody (Cell Signaling Technology; 1:4000), anti-v-ATPase a2 (Abcam; 1:100), or anti-v-ATPase B2 (Abcam; 1:1000) in blocking buffer (5% FCS in 0.02% Triton X-PBS) and Texas Red conjugated goat anti-mouse or rabbit antibody (SouthernBiotech; 1:100) in blocking buffer for 1 h at room temperature in darkness. Coverslips were washed and mounted with DABCO-glycerol medium (Sigma-Aldrich) containing DAPI (1:10,000; Sigma-Aldrich).

### Confocal imaging

Fixed cells were imaged using a Leica TCS SPE confocal laser scanning microscope (Leica Microsystems GmbH) equipped with diode lasers of 405, 488, 532 and 635 nm, using oil immersion objectives (×63, numerical aperture = 1.4). Optical sections were recorded with either two or three scans for each image. ImageJ software was used to analyse the images. Image brightness and contrast was adjusted to the same settings where needed.

### Statistics

All data are presented as means ± SEM. Statistical analysis was performed by using two-sided Student’s *t-*test, and when possible paired testing was applied. P-values of less than 0.05 were considered statistically significant.

## Results

Visualizing proteins with the biarsenical reagent FlAsH-EDT_2_ is based on its high binding affinity to the tetracysteine motif [17]. We predicted three different sites that were remote from either the hydrophobic binding pocket or the two known phosphorylation sites on the extracellular loop, i.e., at amino acid positions 48, 89, and 398, respectively.

**Fig 3:**
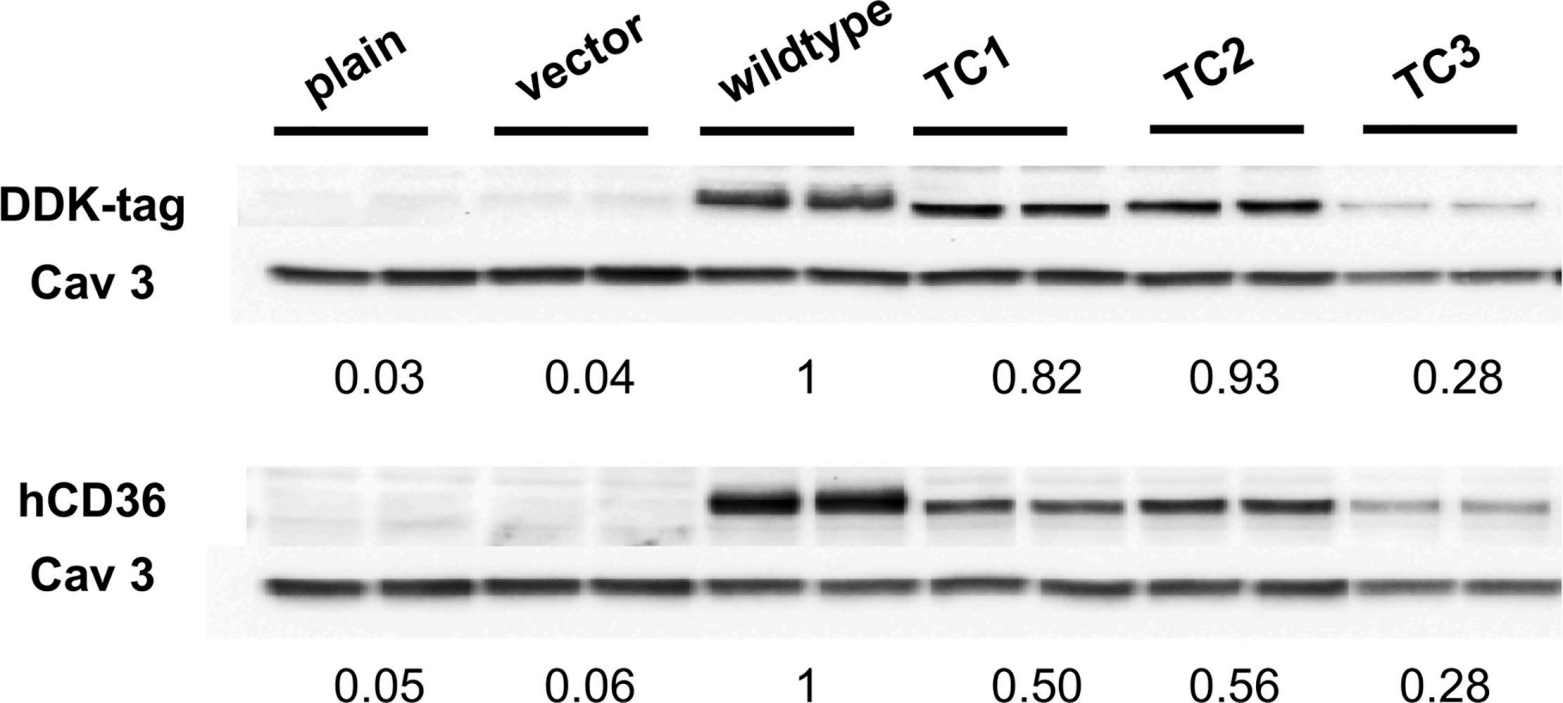
Protein levels as determined by western blotting after lentiviral transduction of hCD36 constructs. Cells were infected with the indicated constructs and cell lysates were prepared for Western blot analysis. Representative immunoblots of 3 independent experiments using hCD36 and its DDK-tag antibodies are shown. Numbers below the blot are means of protein amount as determined by densitometric analysis relative to Caveolin 3. A second normalization was performed by setting the wildtype at 1.

In order to establish stable hCD36-expressing cell lines, mouse HL-1 cardiomyocytes were infected with lentivirus carrying empty vector, wildtype or mutant hCD36, the latter carrying an insertion of the tetracysteine motif at one of the three different sites within the extracellular domain (**Fig 2**). The cells infected with empty lentiviral vector or wildtype hCD36 were taken as controls. To verify hCD36 expression, we used CD36 antibodies that specifically recognize the human but not the mouse protein. In addition, we exploited the vector encoded DDK-tag. Accordingly, cell lysates were analyzed by Western blotting, revealing that neither hCD36 nor DDK-tag was detected in control empty vector, whereas hCD36 wildtype and mutants were detected using both antibodies (**Fig 3**). These results suggested that the corresponding proteins were being appropriately expressed in HL-1 cardiomyocytes.

**Fig 4:**
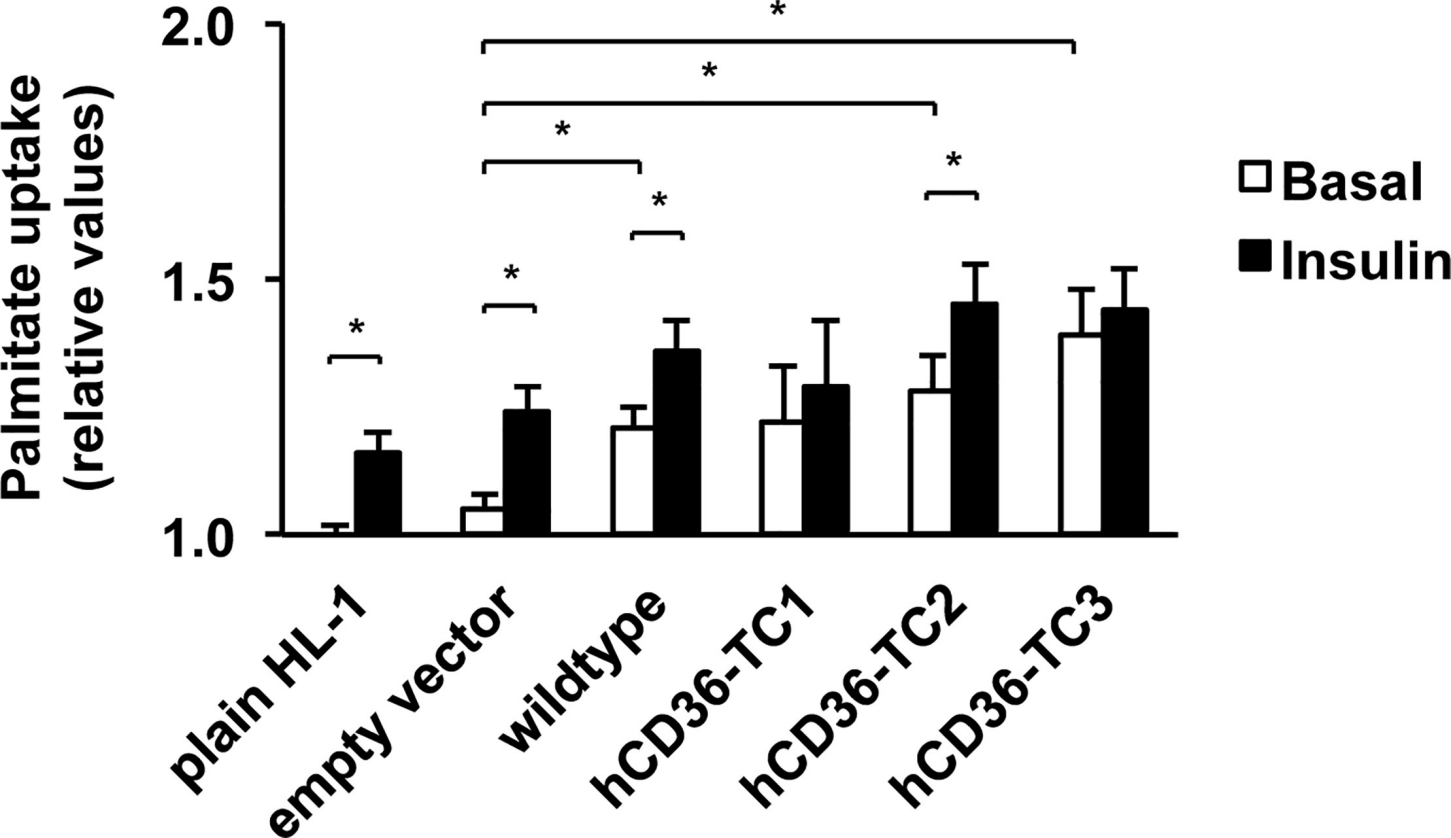
Palmitate uptake after lentiviral transduction of hCD36 constructs under basal and short-term insulin stimulation conditions. Prior to the uptake assay, cells were incubated in serum-free depletion medium for 16 h. Cells were then treated either with or without 200 nM insulin for 30 min in presence of [^14^C] palmitate. Subsequently palmitate uptake was determined by scintillation counting. Values are displayed as mean ± SEM. (n = 3) *p<0.05.

To investigate whether the different mutants still preserve fatty acid transport function, we assessed fatty acid uptake in the absence or presence of insulin. Basal fatty acid uptake was increased 1.2–1.4-fold in both the wildtype hCD36 and two tetracysteine-tagged hCD36 transfected cells (hCD36-TC2 and hCD36TC3) compared to empty vector (**Fig 4**, white bars), indicating increased fatty acid uptake capacity due to the additional hCD36 on top of the endogenous mouse CD36 (mCD36). Short-term insulin stimulation enhanced fatty acid uptake by 1.2-fold in non-transfected HL-1 cells. Similarly, the insulin effect was observed in control empty vector (1.2-fold), wildtype hCD36 (1.1-fold), as well as hCD36-TC2 (1.2-fold), but not in hCD36-TC1and -TC3 (**Fig 4**, black bars). Taken together, based on the enhancement of fatty acid uptake and the retaining of insulin stimulation, the hCD36-TC2 mutant was selected for further studies.

**Fig 5:**
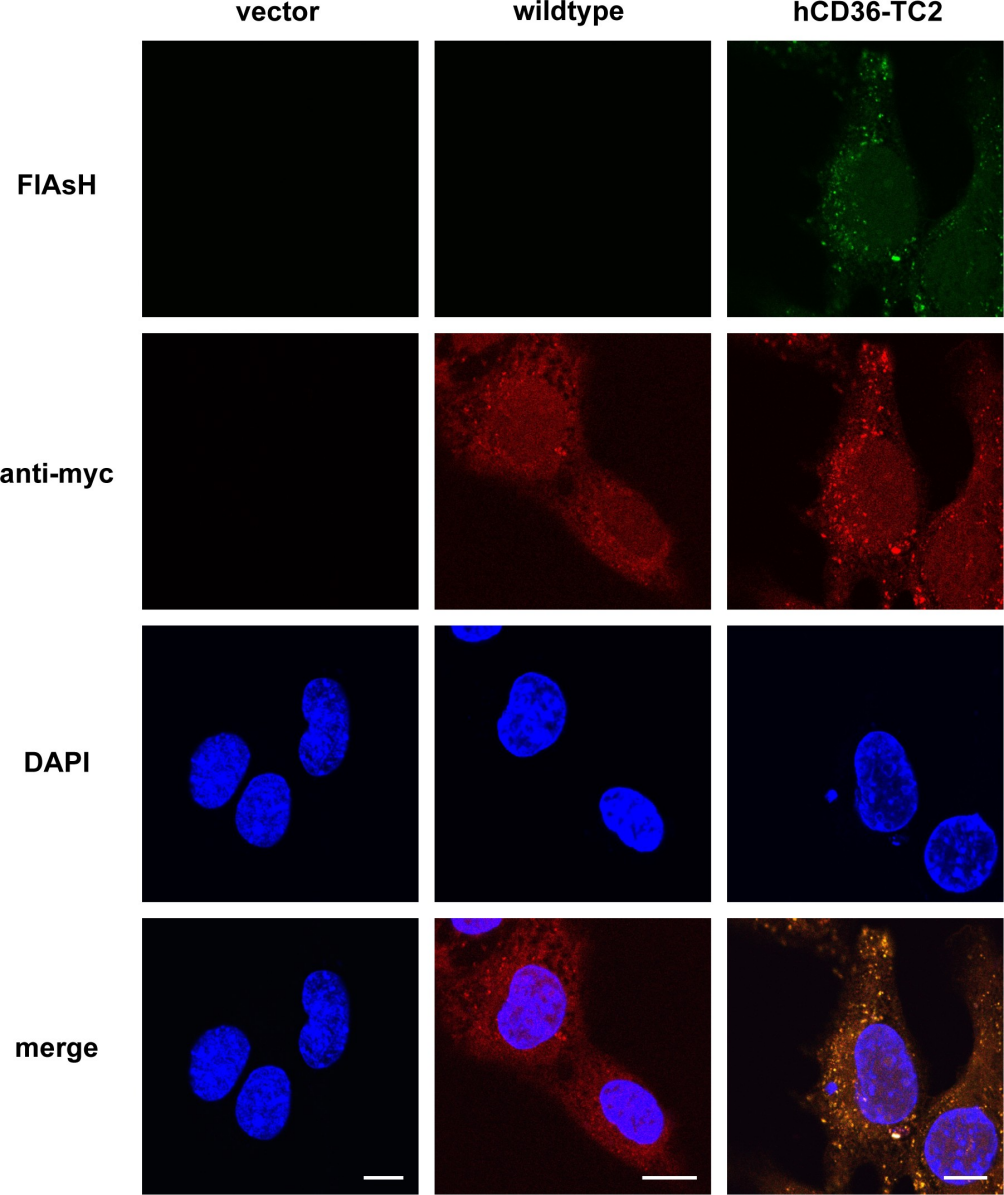
Labelling of tetracysteine-tagged hCD36 and immunofluorescence with anti-myc antibody. Shown are plain HL-1, cells stably overexpressing wildtype of hCD36, and mutant-2, as indicated. Cells were fixed and stained with FlAsH-EDT2 (shown in green). Thereafter hCD36 was immune stained using anti-myc antibody detected by Texas red-labelled secondary antibody (shown in red). Cells were stained with DAPI for visualisation of nuclei (shown in blue). Fluorescent staining was assessed by confocal imaging. Data are representative of three experiments. Images have been adjusted for brightness and contrast for presentation. Scale bars correspond to 10 μm.

We next examined the cellular fluorescent labeling of tetracysteine-tagged hCD36 with FlAsH-EDT_2_ in combination with immunofluorescence by an anti-myc antibody. In cells with the control empty vector, immunofluorescence or FlAsH-EDT_2_ did not result in any fluorescent signals. Immunofluorescence exhibited a speckled pattern in both wild-type hCD36 and hCD36-TC2, as shown by using myc-antibody. A similar speckled pattern from FlAsH-EDT_2_ was seen in hCD36-TC2, not in wild-type hCD36, likely reflecting the binding of tetracysteine motif and FlAsH (**Fig 5,** upper panels). The speckled pattern is in line with endosomal localization. Notably, the speckled pattern of hCD36-TC2 from FlAsH-EDT_2_ co-localized with myc, indicating that the speckles indeed represented hCD36 presence in endosomes (**Fig 5**, lower panels).

**Fig 6:**
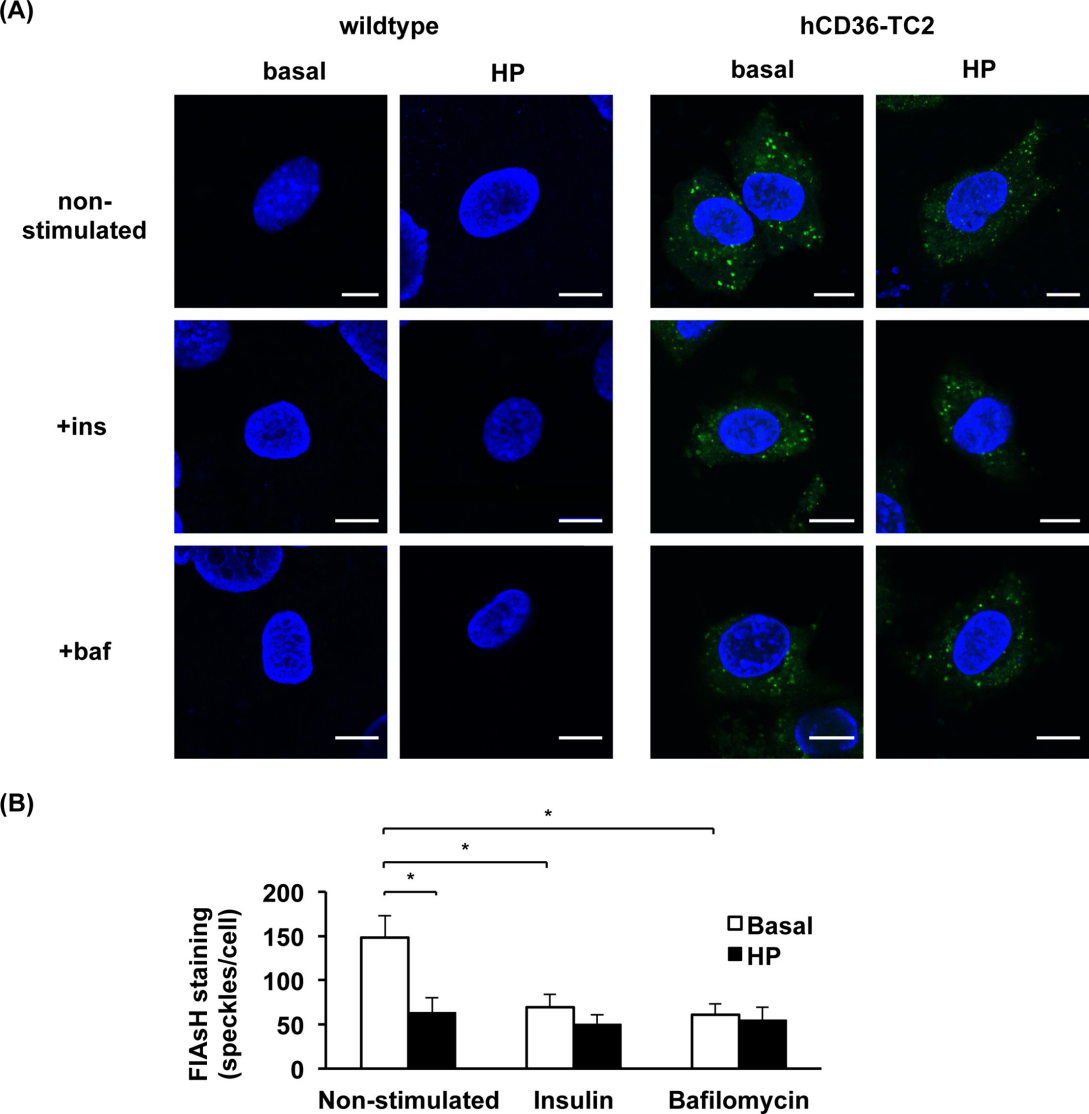
Labelling of wildtype and TC2 mutant hCD36 in basal conditions or upon high-palmitate (HP) treatment. Indicated cells were incubated with serum-starved medium or HP medium for 16 h and thereafter were treated for 30 min with 200 nM insulin or 100 nM bafilomycin. **(A)** Cells were fixed and stained with FlAsH-EDT2 (shown in green). Data are representative of three experiments. Images have been adjusted for brightness and contrast for presentation. **(B)** Quantification of FlAsH-EDT2 stained speckles in hCD36-TC2 expressing cells comparing non-stimulated, insulin, and bafilomycin conditions. Counts are averages of 30 images per condition and are displayed as mean ± SEM. (n = 3) *p<0.05. Scale bars correspond to 10 μm.

We further assessed FlAsH-EDT_2_ staining in both wildtype hCD36 and hCD36-TC2 upon different stimulations. FlAsH-EDT_2_ staining under basal conditions yielded an intracellular speckled pattern. The speckled staining clearly decreased upon both short-term insulin stimulation and bafilomycin treatment (**Fig 6A**). These observations may indicate that in both conditions CD36 translocates to the cell surface, where the signal is more diffuse. Additionally, high-palmitate treatment led to a similar loss of speckles. When this condition was subjected to short-term insulin or bafilomycin stimulation, no further loss of the speckled staining was observed (**Fig 6A**). Quantification of the speckled pattern showed high-palmitate treatment induced a decrease to 50% in endosomal staining compared to basal condition, which also occurred upon short-term stimulations with insulin or bafilomycin. No further decrease was seen upon insulin or bafilomycin treatment if cells were incubated in high-palmitate medium (**Fig 6B**). Thus, wild-type hCD36 and hCD36-TC2 showed similar biological responses in all tested conditions. Therefore, hCD36-TC2 appears to be a suitable construct for further imaging studies.

**Fig 7:**
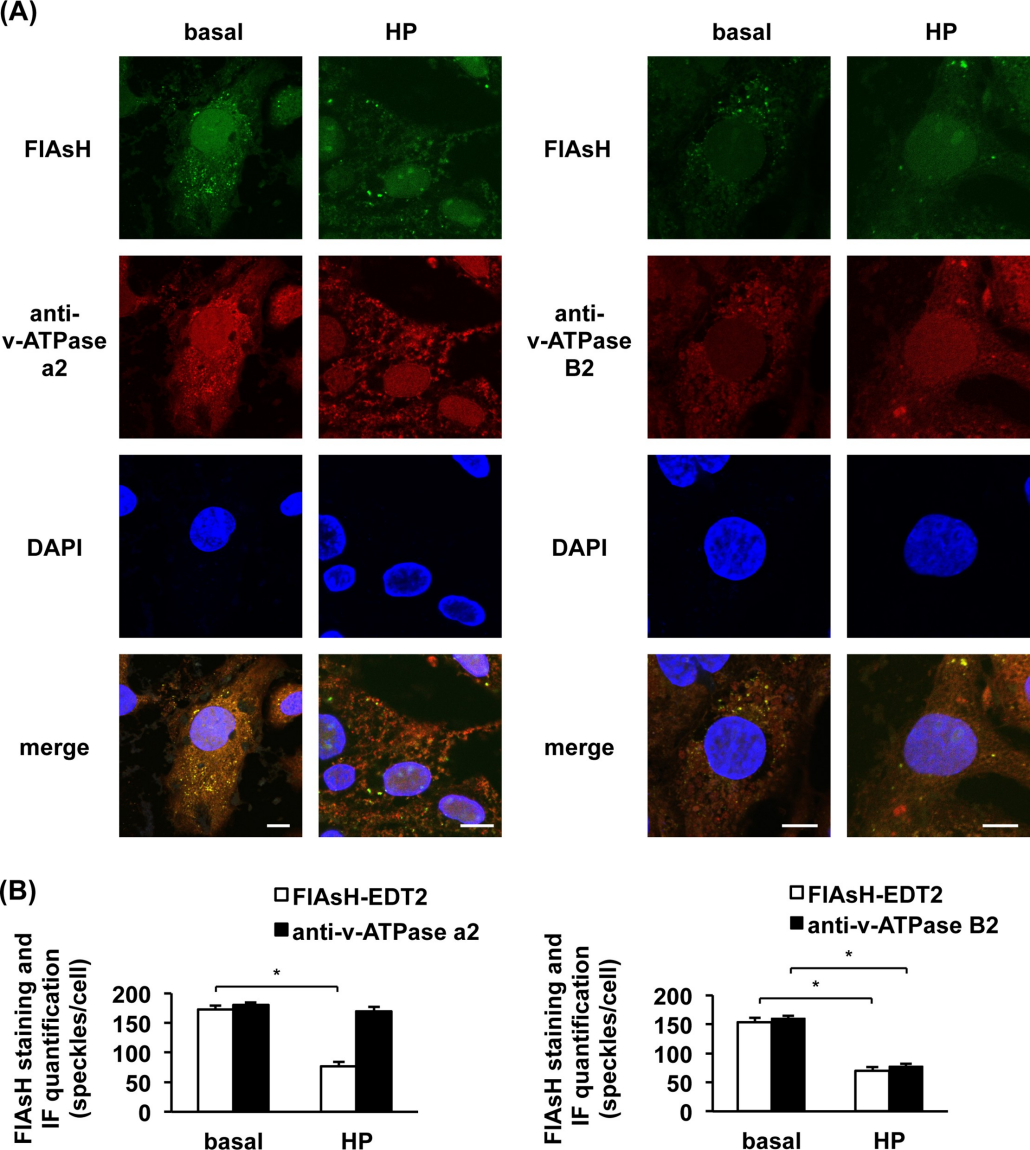
Labelling of hCD36-TC2 in basal conditions or upon high-palmitate (HP) treatment in combination with immune detection of v-ATPase a2 and B2 subunit proteins. Indicated cells were incubated with serum-starved medium or HP medium for 16 h. **(A)** Cells were fixed and stained with FlAsH-EDT2 (shown in green). Thereafter v-ATPase a2 or B2 proteins were immune stained using the respective protein-specific antibodies detected by Texas red-labelled secondary antibody (shown in red). Cells were stained with DAPI for visualisation of nuclei (shown in blue). Fluorescent staining was assessed by confocal imaging. Data are representative of three experiments. Images have been adjusted for brightness and contrast for presentation. **(B)** Quantification of fluorescent speckles for cells as either stained with FlAsH-EDT2 or immune stained with v-ATPase a2 (left) or B2 (right) antibodies in hCD36-TC2 expressing cells. The bar graphs show a comparison of basal condition versus HP-treated cells. Counts are averages of 30 images per condition and are displayed as mean ± SEM. (n = 3) *p<0.05. Scale bars correspond to 10 μm.

Previously, we found that lipid-induced CD36 translocation to the cell surface was due to disassembly of the two sub-complexes V_0_ and V_1_ of v-ATPase and the resulting loss of v-ATPase activity [10]. This conclusion was based on both immunoprecipitation and subcellular fractionation experiments. In order to visualize lipid-induced CD36 relocation and v-ATPase disassembly by microscopy, we combined FlAsH-EDT_2_ staining with immunofluorescence using antibodies directed against either v-ATPase a2 (part of the integral membrane V_0_ sub-complex) or v-ATPase B2 (part of the membrane-associated V_1_ sub-complex). a2 Subunit staining presented as a speckled pattern, co-localizing with FlAsH-EDT_2_ staining in the basal condition. In contrast, the FlAsH-EDT_2_ staining decreased by 60% upon high-palmitate treatment. Yet, the a2 speckled pattern remained unchanged upon high palmitate treatment compared to the basal condition (**Fig 7**). While in the basal condition also the B2 subunit staining was found to co-localize with FlAsH-EDT_2_ staining, upon high-palmitate treatment the number of cytosolic speckles decreased by 50% for B2, similarly as with FlAsH-EDT_2_ (**Fig 7**)_._ These results are again in agreement with translocation of CD36 to the plasma membrane upon exposure to media containing high lipid concentrations. Furthermore, the data are compatible with dissociation of v-ATPase V_1_ from the endosomal membrane bound V_0_ sub-complex by high-palmitate.

## Discussion

Tetracysteine-based labelling of protein has been successfully achieved via biarsenical dyes, such as FlAsH [14–16]. As the tetracysteine sequence, Cys-Cys-Pro-Gly-Cys-Cys, rarely appears in endogenous proteins [14], incorporating the sequence into target proteins generates a small but highly specific target for FlAsH protein labelling. In our current study, we introduced tetracysteine motifs to the extracellular domain of hCD36 at three different positions. If transduced with hCD36-TC2 (carrying the insert at amino acid position 89), HL-1-cardiomyocytes, after selection with antibiotics, showed overexpression of hCD36 in conjunction with enhanced fatty acid uptake and sensitivity to different stimuli, i.e., similar to the wildtype construct. The crystal structure of CD36 was inspected (PDB: 5lgd) to determine the location of the TC2 insertion. Although revealing residue 89 as part of a cell-surface accessible β-sheet, this methodology does not allow drawing conclusions whether glycosylation or phosphorylation could be affected by the TC2 insertion. The other two mutants did not show enhanced fatty acid uptake upon insulin stimulation, which could be due to subtle changes of protein function despite the small tag size. In this respect, it will be interesting to determine if the mutations impact CD36 co-localization or expression of other binding partners (e.g. TLR and caveolin-3) that are known to impact the signaling and localization of the protein [20, 21].

We also combined FlAsH-EDT_2_ labelling with immunofluorescence staining of the myc epitope encoded by hCD36-TC2. The fluorescent staining pattern was congruent, suggesting that the speckled pattern resulting from FlAsH-staining indeed represents CD36. The speckled pattern is in agreement with the expected endosomal localization of CD36, as verified by co-staining with v-ATPase a2-subunit (Fig 7; [10]). Furthermore, we demonstrated that different stimuli (insulin, bafilomycin, and palmitate) triggered the disappearance of hCD36-TC2 from the endosomal compartment in keeping with the known translocation of CD36 to the cell surface (as previously shown by subcellular fractionation [4], cell surface biotinylation [19], ELISA-based colorimetry [18], and 2-photon microscopy [13]). These data also strongly argue against artefacts as being responsible for the observed patterns. However, we could not visualize the increased surface localization of CD36, which may be due to a more diffuse staining pattern after translocation to the membrane. Collectively, our findings suggest hCD36-TC2 as a potential candidate to label with biarsenical dyes. Notably, the availability of lentiviral vectors encoding tetracysteine-tagged CD36 should facilitate evaluation of the method in wide variety of cultured cell types in addition to HL-1 cardiomyocytes.

A major advantage of the tetracysteine motif insertion is the small tag size and the flexibility that staining with biarsenical dyes can offer for downstream applications. Despite the obvious merits, also a few disadvantages that may restrict the use of FlAsH-EDT_2_ and thus may affect the application. Firstly, in our hands the non-specific binding with excess dyes gave a relatively high background, which we needed to remove with high-concentrations of EDT, followed by multiple washing steps with HBSS/glucose as compared to the standard protocol [22]. Perhaps, the requirement of washing steps is also cell-dependent, which has to be tested experimentally. Secondly, in order to selectively study cell surface-localized CD36, membrane-impermeable biarsenical dyes are desirable. Yet, the commercially available FlAsH-EDT_2_ and ReAsH-EDT_2_ both are membrane-permeable, and thus can stain the entire cellular CD36 protein population. A sulfonated derivative of FlAsH (sFlAsH), which has been described as membrane-impermeable, is not commercially available, and the synthesis of such compound is beyond our expertise [17]. An alternative method to exclusively label extracellular tetracysteines is to pre-incubate FlAsH-EDT_2_ with DMPS (dimercaptopranesulfonate sodium salt, Sigma-Aldrich). This compound exchanges the membrane-permeable EDT for the charged DMPS and has been applied successfully in tetracysteine-tagged proteins in intact cells [22]. Following this protocol, we made several attempts to exclusively label surface CD36. However, this procedure proved only partially effective. Despite improved surface CD36 labelling, some intracellular CD36 staining remained.

Another technology similar to FlAsH-EDT_2_ uses small fluorogen activating peptide (FAP), a novel fluorescent biosensor that can be turned on and off by adding or removing fluorogen, or by changing the signal wavelength through substitution of one fluorogen for another [23, 24]. This technology is particularly useful in trafficking studies since cell surface labeling is feasible by incubating mutant FAP-protein-transfected cells with a membrane impermeable fluorogen. However, the relative large size of the FAP-tag (25 kDa) limits its fusion site to the protein N-terminus, and therefore is not suitable for CD36 trafficking studies, as both C- and N-terminals are localized intracellularly, i.e., not allowing the binding of FAP-tag to impermeable fluorogen. The combination of those two techniques, offering a relative small size of the tag and an impermeable fluorogen, we would judge as the ideal approach for CD36 trafficking studies. Commercial availability of (novel) membrane impermeable biarsenical dyes could resolve this issue in short term.

We previously identified v-ATPase as a mediator of lipid-induced CD36 relocation to the sarcolemma and the resulting lipid accumulation and development of cardiac insulin resistance [10]. Assembly/disassembly of the two v-ATPase sub-complexes, V_1_ and V_0_, is the main mechanism of regulation of v-ATPase activity in yeast [25]. This mechanism is sensitive to glucose concentrations, which favour assembly and, hence, v-ATPase activation [26]. Vice versa, in the mammalian heart, v-ATPase appears to be regulated by assembly/disassembly, with high lipid concentrations inducing the dissociation of the V_1_ sub-complex, whereas the V_0_ sub-complex remained integral to the endosomal membrane [10]. This novel mechanism was revealed using both immunoprecipitation and subcellular fractionation methods [10]. In the present study, we further investigated lipid-induced v-ATPase disassembly in cardiomyocytes by direct microscopic observation. Indeed, the combination of FlAsH-EDT_2_ staining and immunofluorescence by antibodies against subunits of the V_1_ and V_0_ sub-complexes is in line with our earlier conclusion that lipid induced dissociation and migration of V_1_ into the cytoplasm is concomitant to increased CD36 translocation to the plasma membrane (**Fig 7**).
